# Trends and outcomes in the utilization of laparoscopic appendectomies in a low-income population in Taiwan from 2003 to 2011

**DOI:** 10.1186/s12939-015-0248-x

**Published:** 2015-10-24

**Authors:** Kai-Biao Lin, K. Robert Lai, Nan-Ping Yang, Ke-Shou Wu, Hsien-Wei Ting, Ren-Hao Pan, Chien-Lung Chan

**Affiliations:** School of Computer & Information Engineering, Xiamen University of Technology, Xiamen, 361024 China; Department of Computer Science and Engineering, Yuan Ze University, Taoyuan, 32003 Taiwan; Management Center, Keelung Hospital, Ministry of Health and Welfare, Keelung, 20147 Taiwan; Institute of Public Health, National Yang-Ming University, Taipei, 11221 Taiwan; Department of Information Management, Yuan Ze University, Taoyuan, 32003 Taiwan; Department of Neurosurgery, Taipei Hospital, Taipei, 10002 Taiwan; Innovation Center for Big Data and Digital Convergence, Yuan Ze University, Taoyuan, 32003 Taiwan

**Keywords:** Appendicitis, Epidemiology, Laparoscopic appendectomy, Open appendectomy, Low-income population, Socioeconomic status

## Abstract

**Background:**

Numerous epidemiological studies have compared outcomes between laparoscopic appendectomies (LA) and open appendectomies (OA); however, few studies have assessed the efficacy of LA specifically in a low-income population (LIP).

**Methods:**

We analyzed the trends in the utilization and outcomes of LA versus OA in an LIP in Taiwan using data from the National Health Insurance (NHI) Research Database.

**Results:**

Steady temporal growth trends were observed for the patients who underwent LA in both the LIP and general population (GP); however, in each study year, the proportion of LIP patients who underwent LA was lower than the proportion of GP patients who underwent the procedure. The LIP patients were more susceptible to payment policies than the GP patients; thus, more attention should be paid to vulnerable patient populations when formulating and revising NHI payment policies. Compared with OAs, LAs were associated with a slightly higher rate of routine patient discharges and a lower rate of in-hospital complications (1.48 % vs. 3.76 %, *p* < 0.05). The rate of readmission for complications was lower in patients after LA than in patients after OA (1.64 % vs. 3.89 %, *p* < 0.05). The overall case-fatality rate of LIP patients who underwent LA was lower than that of those who underwent OA. LA was correlated with a significantly shorter length of hospital stay (LOS) compared with OA (3.80 ± 0.08 vs. 5.51 ± 0.11, *p* < 0.05). The average hospital cost for LA was slightly less than that for OA (1178 ± 13 vs. 1191 ± 19 USD, *p* < 0.05). A higher percentage of patients who underwent OA required an LOS longer than 14 days compared to patients who underwent LA (7.73 % vs. 1.97 %, *p* < 0.05). Regarding hospital costs and LOS, LA showed significant advantages over OA in the subpopulations of male patients, patients 45 years old and older, patients with Charlson Comorbidity Index (CCI) scores of two or more, and patients with complicated cases of appendicitis.

**Conclusion:**

The LIP patients benefited more from the LA approach than the OA approach in the treatment of appendicitis, especially regarding LOS, in-hospital complications, in-hospital mortality, and routine discharge rates.

## Introduction

The low-income population (LIP) is more subject to serious disease than the general population (GP) in Taiwan [1, 2]. Some previous studies [3–5] have shown that lower socioeconomic status has been linked to impaired access to surgical care, and delay in treatment is a strong risk factor for perforation during acute appendicitis. Furthermore, these findings suggest that patients with no insurance or public insurance have increased appendiceal perforation rates compared with patients with private insurance. In our previous study [6], we found that the overall incidence of appendicitis in the LIP was substantially higher than that in the GP (139.54 vs. 102.41 per 100,000 per year, *p* < 0.05). The mean length of hospital stay (LOS) in the LIP patients was longer than that in the GP patients (5.34 ± 0.09 vs. 4.72 ± 0.01 days, *p* < 0.05). Furthermore, the overall case-fatality rate of appendectomy in the LIP was higher than that in the GP (0.41 % versus 0.12 %, *p* < 0.05) [6]. Based on these findings, we confirmed that having a lower socioeconomic status results in a significant negative impact on the occurrence and treatment of appendicitis, as well as on the outcomes of appendectomies. Expanding upon our previous findings and using data from the National Health Insurance Research Database (NHIRD) from 2003 to 2011, the objective of this study was to examine trends in the utilization and outcomes of laparoscopic appendectomies (LAs) versus open appendectomies (OAs) in the LIP.

Since its first description by Semm, who used laparoscopy to remove the appendix [7], LA has become an increasingly popular treatment modality because it allows for better visualization, fewer wound infections, less postoperative pain, shorter hospital stays, and an earlier return to daily activities compared with OA [8–12]. However, LA is not routinely performed for appendicitis because the costs associated with that procedure are higher than those for OA [10]. The debate over LA versus OA has remained lively [13]; some studies [14–16] have addressed whether LA is feasible for patients with perforated appendicitis, and some surgeons expressed doubts concerning the utilization of laparoscopy for appendectomy. Although numerous epidemiological studies have compared the outcomes between LA and OA [17–19], few studies have assessed the efficacy of LA and OA in an LIP [6, 20]. Thus, conducting in-depth research and analyses of the effect of LA and OA on LIP patients is necessary; such studies may lead to treatment suggestions for medical research institutions and may assist surgeons in making decisions concerning the management of LIP patients with appendicitis and concerning the judicious use of LA.

## Materials and methods

### Data source

Taiwan launched the single-payer National Health Insurance (NHI) program in 1995. By 2000, the NHI coverage rate had expanded to 96.16 % of the Taiwanese population, and by 2011, coverage had reached 99.88 %. All eligible enrollees can access health care services at most clinics and hospitals by making a small copayment [21]. The National Health Insurance Bureau (NHIB) established a nationwide research database, including nationwide population-based data with high quality control and representation. The NHIRD includes various data subsets, such as inpatient expenditures by admissions (DD), details of inpatient orders (DO), ambulatory care expenditures by visits (CD), and details of ambulatory care orders (OO). In this study, the DD dataset was used for further analysis.

### Data protection and permission

The personal information of all subjects was encrypted with a double scrambling protocol for research purposes to protect patient privacy. All researchers who wish to use the NHIRD and its data subsets are required to sign a written agreement declaring that they have no intention of obtaining information that could potentially violate the privacy of the patients or care providers. This study was approved by the Institutional Review Board (IRB) of the Taoyuan General Hospital, which has been certified by the Ministry of Health & Welfare of Taiwan (IRB Approval Number: TYGH103015), and the protocol was evaluated by the National Health Research Institutes (NHRI), which consented to this planned analysis of the NHIRD (Agreement Numbers: NHIRD-103-160 and NHIRD-104-081).

### Data definition

To investigate the incidence of appendicitis in Taiwan in this study, we used the diagnosis codes from the International Classification of Diseases, Ninth Revision, Clinical Modification (ICD-9-CM). Appendicitis comprised the diagnosis codes of 540 (acute appendicitis), 541 (appendicitis, unqualified), 542 (other appendicitis), and 543 (other disease of the appendix) [6]. Patients who underwent OA were identified by the ICD-9-CM procedure codes 47.0 (appendectomy, excludes incidental) and 47.09 (other appendectomy), and patients who underwent LA were identified by the ICD-9-CM code 47.01 (laparoscopic appendectomy). Complicated appendicitis was defined as appendicitis with a perforation, an abscess formation, or peritonitis. The case-fatality rate was defined as the percentage of patients with appendectomy who died during hospitalization.

### Classification of LIP and GP

To evaluate the effects of socioeconomic status, the enrolled subjects were divided into GP and LIP groups based on whether they satisfied the criteria of Taiwan’s Social Assistance Act and whether they were registered in Taiwan’s NHI database. Low-income households were defined as those with an average per-person gross monthly income of less than the monthly minimum living expense standard of that residence region. The minimum living expense standard was defined as 60 % of the average monthly disposable income for each region. The family property was not permitted to exceed a certain amount, as determined by the central or municipal authorities in the corresponding year [22]. This subpopulation was recorded as the fifth class insured in Taiwan’s NHI database [21]. The GP refers to individuals who are not classified as LIP.

### Measured outcomes

#### Length of hospital stay

The period between admission and discharge was defined as the LOS (measured in days). The LOS was recorded as 1 day for patients discharged on the same day that they were admitted to the hospital [10].

#### Hospital costs

Hospital costs were calculated by summing all of the items enumerated in the hospital discharge summary, including operation-associated costs and ward costs. The operation-associated costs included anesthesia and surgery fees, as well as the cost of medical supplies used during the operation. The surplus costs were classified as ward costs. The costs expressed in this study are in U.S. dollars (USD). In 2007, one USD dollar was equivalent to approximately 32.64 Taiwan dollars [10].

#### In-hospital complications

We examined all-cause non-fatal in-hospital morbidity data based on ICD-9 codes. Complications were grouped into 8 categories (mechanical wound complications, infections, urinary complications, pulmonary complications, gastrointestinal complications, cardiovascular complications, systemic complications, and complications during procedures; Table 1). Because the DD dataset of the NHIRD only contains inpatient data, complications that occurred after hospital discharge were not included in our analysis.

**Table 1:** ICD-9 codes for postoperative in-hospital complications

Complications	ICD-9 codes
Mechanical wound complications	998.12, 998.13, 998.3, 998.6, and 998.83
Infections	998.5, 998.51, and 998.59
Urinary complications	997.5
Pulmonary complications	512.1, 518.4, 518.5, and 997.3
Gastrointestinal complications	997.4
Cardiovascular complications	415.11, 997.02, 997.1, 997.2, and 997.79
Systemic complications	998.0 and 998.89
Complications during procedure	998.11, 998.2, and 998.4

#### In-hospital mortality

In-hospital mortality was defined as the patients with appendectomy who died during hospitalization. Because the NHIRD DD dataset only contains inpatient data, deaths that occurred after hospital discharge were not included in our analysis.

#### Routine discharge rate

The NHIRD provides information about the patient’s discharge status (1, treated and discharged; 2, remaining in the hospital; 3, changed to outpatient treatment; 4, dead; 5, discharged against medical advice; 6, referred to another facility; 7, identity changed; 8, left without being discharged; 9, suicide; 0, other; and A, died after discharge). The patients were grouped into routine discharge (1, 3) or non-routine discharge groups (0, 2, 4–9, and A).

#### Readmission for complications

Readmission for complications was defined as readmission with the diagnosis of a commonly encountered postoperative complication within 1 month following an appendectomy (Appendix B in [23]).

### Statistical analysis

For analysis, the following descriptive statistics were generated: the baseline characteristics were shown by the number of cases, percentages, annual incidence rates (per 100,000 people), and 95 % confidence intervals (CI) for the estimated rates. Pearson’s chi-squared (*χ*^2^) test was used to evaluate the statistical significance between the non-continuous variables of LIP and GP, and analysis of variance (ANOVA) was used to describe and compare continuous variables among different subgroups. The significance level was set at *p =* 0.05. Multiple linear regression models were used to identify the linear effects of variables separately for LA and OA. To reduce the impact of outlier data on the means of LOS and hospital costs, we excluded 1 % of the maximum values and 1 % of the minimum values from the raw data. All statistical analyses were performed using the Statistical Package for Social Sciences for Windows (SPSS for Windows Version 18.0).

## Results

From 2003 to 2011, 2916 patients from the LIP were diagnosed with appendicitis. Among these patients, 49.90 % were male, 49.90 % were female, and the remaining 0.20 % of the patients had missing gender information. The overall incidence of appendicitis for the LIP was 139.54 per 100,000 per year (95 % CI: 132.22–146.85). A total of 2687 patients underwent an appendectomy. Among them, 2533 patients (94.27 %) were diagnosed with acute appendicitis at discharge, 75 patients (2.79 %) were diagnosed with the ICD codes 541–543 (i.e., unqualified appendicitis, other appendicitis or other disease of the appendix) at discharge, and the remaining 79 patients (2.94 %) were recorded without the diagnosis of appendicitis, indicating that they may have been misdiagnosed.

**Fig. 1:**
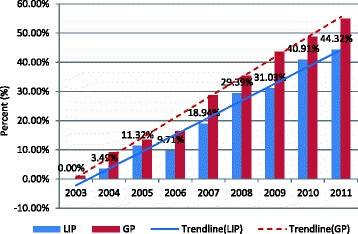
Temporal trends in the proportion of selection of LA by LIP and GP patients in Taiwan, 2003–2011

**Fig. 2:**
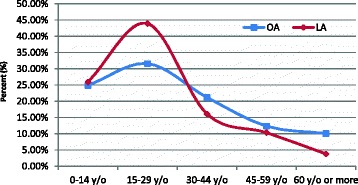
Age-specific proportions of the patients who underwent LA and OA, 2003–2011

During the observation period, 2077 patients underwent OA, and 610 patients underwent LA; in no case was the operative procedure converted from LA to OA. The general percentage of OA and LA revealed that more LIP patients underwent OA than LA (77.30 % vs. 22.70 %, *p* < 0.5). However, as shown in Fig. 1, the percentage of patients who underwent LA increased in the LIP and GP from 2003 to 2011. For example, in 2003, the percentage of patients in the LIP who underwent LA was 0 %, and the value increased to 44.32 % by the end of the 9-year study period in 2011. In the GP, more than half (51.20 % for LA vs. 48.80 % for OA, *p* < 0.5) of the patients underwent LA in 2010, and the percentage increased by 2011 (54.94 % for LA vs. 45.05 % for OA, *p* < 0.5). Although the temporal trends for the percentage of patients in the LIP who underwent LA exhibited steady growth, the slope of the trend line for the proportion of LIP patients who underwent LA (0.058, *p* < 0.05) was lower than that for GP patients (0.069, *p* < 0.05), which indicated that the upward trend of the LIP was lower than that of the GP. In other words, the growth trend of the proportion of LIP patients who underwent LA was lower than that of the GP patients (Fig. 1). The age-specific percentage of patients displayed a similar pattern for both OA and LA; the highest percentage was observed in the 15- to 29-year-old age group, and the lowest percentage was in patients aged 60 years and older (Fig. 2).

As shown in Table 2, 3.91 % of the LIP patients who underwent an appendectomy exhibited a Charlson Comorbidity Index (CCI) score of one, and 2.20 % exhibited a CCI score of two or more. Patients with a CCI score of more than one were more likely to undergo OA than LA (6.79 % for OA vs. 4.18 % for LA, *p* = 0.072). The percentage of patients residing in urban areas (76.14 %) was higher than the percentage residing in suburban (21.99 %) or rural (1.86 %) areas. The proportions of patients who underwent LA were 25.07 % in urban areas, 16.07 % in suburban areas, and 4.00 % in rural areas. This finding indicated that patients residing in urban or suburban areas were more likely to choose LA than patients residing in rural areas. A higher percentage of LIP patients were hospitalized in regional hospitals (52.66 %) than in medical centers (24.97 %) or district hospitals (22.40 %). However, most of the patients who underwent LA were hospitalized in regional hospitals (44.43 %) or medical centers (41.80 %) rather than district hospitals (12.77 %). In medical centers, the proportion of LA was 38.00 %, which was higher than the proportions in regional hospitals (19.15 %) and district hospitals (13.95 %).

**Table 2:** Demographic characteristic of patients with appendicitis in Taiwan from 2003 to 2011

Variable	All (*n* = 2687)		LA (*n* = 610)		OA (*n* = 2077)		*P* value
	*n*	%	*n*	%	*n*	%	
Gender							0.349
Female	1335	49.68 %	318	52.13 %	1017	48.96 %	
Male	1352	50.32 %	292	47.87 %	1060	51.04 %	
Age stratum							<0.001
0–14 y/o	674	25.08 %	158	25.90 %	516	24.84 %	
15–29 y/o	924	34.39 %	268	43.93 %	656	31.58 %	
30–44 y/o	538	20.02 %	98	16.07 %	440	21.18 %	
45–59 y/o	319	11.87 %	63	10.33 %	256	12.33 %	
60 y/o or more	232	8.63 %	23	3.77 %	209	10.06 %	
Complicated appendicitis							0.003
No	2010	74.80 %	466	76.39 %	1544	74.34 %	
Yes	678	25.23 %	144	23.61 %	534	25.71 %	
CCI score^a^							0.072
0	2523	93.90 %	587	96.23 %	1936	93.21 %	
1	105	3.91 %	14	2.30 %	91	4.38 %	
≥ 2	59	2.20 %	9	1.48 %	50	2.41 %	
Hospital level							<0.001
District Hospital	602	22.40 %	84	13.77 %	518	24.94 %	
Regional Hospital	1415	52.66 %	271	44.43 %	1144	55.08 %	
Medical Center	671	24.97 %	255	41.80 %	416	20.03 %	
Area level							<0.001
Urban	2046	76.14 %	513	84.10 %	1533	73.81 %	
Suburban	591	21.99 %	95	15.57 %	496	23.88 %	
Rural	50	1.86 %	2	0.33 %	48	2.31 %	

*OA* open appendectomy, *LA* laparoscopic appendectomy, *CCI* charlson comorbidities index

^a^The CCI, which was developed by Charlson et al. [29], is a validated method for classifying comorbid conditions that might alter the risk of mortality for use in longitudinal studies. The index score is the sum of the assigned weights and represents a measure of the burden of comorbid disease

LA was associated with a higher rate of routine patient discharges (99.67 % after LA vs. 98.07 % after OA, *p* < 0.05). The rate of readmission for complications was higher in patients who underwent OA than in patients who underwent LA (3.89 % vs. 1.64 %, *p* < 0.05). The overall case-fatality rate of patients who underwent LA was 0 %, but the case-fatality rate of patients who underwent OA was 0.53 %. Of these patients, 5 (45.45 %) were male and 6 (54.55 %) were female; on average, the patients were 64.6 ± 14.3 years old, and 4 patients (36.36 %) had a CCI score of one or more. LA was also associated with a lower rate of in-hospital complications than OA (1.48 % after LA, 3.76 % after OA, *p* < 0.05). Systemic complications account for 87.36 % of the total number of complications in patients; none of the LIP patients had mechanical wound complications, infections, urinary complications, or cardiovascular complications in the hospital after LA or OA (Table 3).

**Table 3:** Characteristics of in-hospital complications, in-hospital mortality, rate of routine discharge and readmission for complications

Variables	All (*N*, %)	Method of appendectomy (*N*, %)		*P* value
		LA	OA	
In-hospital mortality	11 (0.41 %)	0 (0.00 %)	11 (0.53 %)	<0.001
Rate of routine discharge	2645 (98.44 %)	608 (99.67 %)	2037 (98.07 %)	<0.001
Readmission for complications^a^	92 (3.42 %)	10 (1.64 %)	82 (3.95 %)	<0.001
In-hospital complications	87 (3.24 %)	9 (1.48 %)	78 (3.76 %)	<0.001
Mechanical wound complications	0 (0.00 %)	0 (0.00 %)	0 (0.00 %)	1.000
Infections	0 (0.00 %)	0 (0.00 %)	0 (0.00 %)	1.000
Urinary complications	0 (0.00 %)	0 (0.00 %)	0 (0.00 %)	1.000
Pulmonary complications	2 (0.07 %)	0 (0.00 %)	2 (0.10 %)	0.111
Gastrointestinal complications	6 (0.22 %)	1 (0.16 %)	5 (0.24 %)	0.224
Cardiovascular complications	0 (0.00 %)	0 (0.00 %)	0 (0.00 %)	1.000
Systemic complications	76 (2.83 %)	7 (1.15 %)	69 (3.32 %)	<0.001
Complications during procedure	3 (0.11 %)	1 (0.16 %)	2 (0.10 %)	0.015

^a^Readmission for complications was defined as readmission with the diagnosis of a commonly encountered postoperative complication within 1 month after an appendectomy

**Fig. 3:**
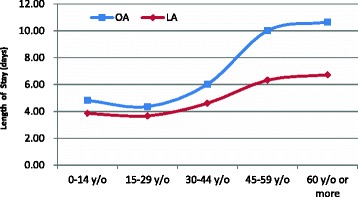
Mean length of hospital stay according to age group in Taiwan, 2003–2011

**Fig. 4:**
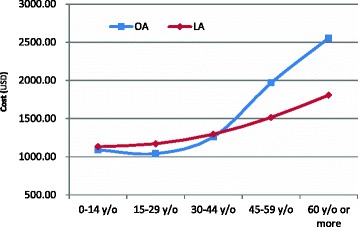
Average hospital cost according to age group in Taiwan, 2003–2011

**Fig. 5:**
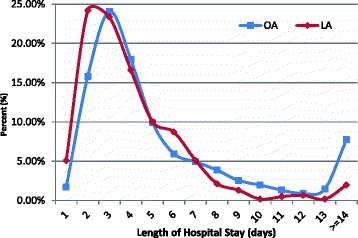
Frequency distribution of length of hospital stay for patients after OA and LA, 2003–2011

From 2003 to 2011, the mean LOS was 5.15 ± 0.09 days for the patients who underwent an appendectomy, and the average hospital cost for patients who underwent appendectomy was 1188 ± 15 USD. Table 4 reveals that LA was correlated with a significantly shorter LOS compared with OA (3.80 ± 0.08 vs. 5.51 ± 0.11, *p* < 0.05); however, the average hospital cost for LA and OA was comparable (1178 ± 13 vs. 1191 ± 19 USD, *p* < 0.05). The age-specific hospitalization times of OA and LA demonstrated that the mean LOS of patients who underwent LA was shorter than those that underwent OA in all age groups. Particularly in the 45- to 59-year-old and 60 years and older age groups, the mean LOS for patients who underwent LA was 3.68 days, which was 3.91 days shorter than that for patients who underwent OA, respectively (Fig. 3). The age-specific average hospital cost exhibited a similar pattern between OA and LA, and the hospital cost increased with age. However, the hospital cost for patients who underwent LA was higher than that for patients who underwent OA in the 0- to 44-year-old age group, but it was lower for patients aged 45 years and older (Fig. 4). The distribution of the mean LOS for patients who underwent LA or OA is displayed in Fig. 4; in most cases, the LOS ranged from 2 to 7 days (87.99 % for LA and 78.59 % for OA, *p* < 0.05). In addition, after OA, a higher percentage of patients had a mean LOS greater than 14 days compared with the LOS for patients following LA (7.73 % for OA vs. 1.97 % for LA, *p* < 0.05) (Fig. 5).

**Table 4:** Medical utilization of appendectomy in Taiwan by operation type, 2003–2011

Operation type	Summed cases 2003–2011 (%)	LOS (days)	Cost (USD)
		Mean ± SE	Mean ± SE
OA	77.30 %	5.51 ± 0.11	1191 ± 19
LA	22.70 %	3.80 ± 0.08	1178 ± 13
ANOVA test		*p = 0.000*	*p = 0.000*

To reduce the impact of outlier data on the means of LOS and hospital cost, we excluded 1 % of the maximum values and 1 % of the minimum values from the raw data

*OA* open appendectomy, *LA* laparoscopic appendectomy

Table 5 shows differences in the adjusted costs and LOS between the LIP and GP patients, as stratified by various determinants. The coefficients in the multiple linear regression models represent the differences in the specific outcomes between the target and the reference groups [10]. For example, the cost for male patients who underwent LA was higher by 74.8 ± 2.6 USD (*p* = 0.099) than the cost for female patients who underwent LA. After multivariate adjustment, we observed that the mean cost and LOS for male patients were higher than those for female patients in both LA and OA groups, but the magnitudes of difference for the cost and LOS in the LA group were significantly lower than those in the OA group. The cost and LOS increased with age in both the LA and OA groups. Meanwhile, the magnitudes of difference for the cost and LOS were significantly lower in the LA group than in the OA group in patients aged 45 to 59 years and 60 years and older. The cost and LOS increased for patients with CCI scores of one or more in both the LA and OA groups, and the corresponding values increased as the CCI score increased for the OA group; intriguingly, however, the cost and LOS for the patients with CCI scores of two or more were lower than those for patients with a CCI score of one in the LA group. We also observed that the cost and LOS for complicated appendicitis were higher than those for uncomplicated appendicitis, but the magnitudes of difference in the LA group were significantly lower than those in the OA group. In addition, the costs for regional hospitals and medical centers were slightly higher than those for district hospitals in the LA group, but the cost for medical centers was significantly higher than those for regional and district hospitals in the OA group. The costs in suburban and rural areas were lower than those in urban areas in both the LA and OA groups, but the magnitudes of difference for cost and LOS in the LA group were significantly lower than those in the OA group. Thus, the magnitudes of difference for cost and LOS for most of the variables in the LA group were lower than those in the OA group, indicating that these variables had a greater impact on costs and LOS for OA patients than for LA patients.

**Table 5:** Multiple linear regression analysis of determinants of hospital costs (USD) and length of hospital stay by laparoscopic appendectomy and open appendectomy

Stratified variables	Hospital cost (USD) (Coefficient ± SE)		LOS (days) (Coefficient ± SE)	
	LA	OA	LA	OA
Gender				
Male vs. female	74.8 ± 2.6	346.9 ± 2.3^***^	0.62 ± 0.02	1.59 ± 0.01^***^
Age stratum (vs. 0–14 y/o)				
15–29 y/o	38.7 ± 3.3	−50.4 ± 3.9	−0.20 ± 0.03	−0.47 ± 0.02
30–44 y/o	164.4 ± 7^*^	168.1 ± 5.2	0.75 ± 0.06	1.19 ± 0.02^**^
45–59 y/o	383.8 ± 10.1^***^	876.4 ± 8.0^***^	2.47 ± 0.08^***^	5.19 ± 0.03^***^
60 y/o or more	676.5 ± 25.5^***^	1464.0 ± 9.5^***^	2.87 ± 0.20^**^	5.81 ± 0.04^***^
CCI score (vs. 0)				
1	976.3 ± 38.4^***^	1092.9 ± 19.4^***^	7.61 ± 0.30^***^	4.82 ± 0.08^***^
≥ 2	783.0 ± 59.5^***^	1878.0 ± 34.6^***^	2.74 ± 0.47^*^	6.76 ± 0.14^***^
Complicated appendicitis				
Yes vs. none	368.5 ± 4.3^***^	784 ± 3.7^***^	2.49 ± 0.03	4.58 ± 0.02^***^
Hospital level (vs. District Hospital)				
Regional Hospital	39.7 ± 4.2	65.1 ± 2.7	−0.27 ± 0.03	−0.51 ± 0.01
Medical Center	17.1 ± 4.4	527.2 ± 5.6^***^	−0.88 ± 0.03	0.68 ± 0.02
Area level (vs. Urban)				
Suburban	−18.2 ± 6.4	−217.8 ± 4^*^	0.19 ± 0.05	−0.60 ± 0.02
Rural	−44.9 ± 280.2	−463.8 ± 38.8	−0.23 ± 2.17	−1.77 ± 0.16

Multiple linear regression was conducted after adjustment for gender, age, comorbidities, complicated appendicitis, hospital level, and area level but not the target variable

*OA* open appendectomy, *LA* laparoscopic appendectomy, *CCI* charlson comorbidity index, *SE* standard error

^*^ *p* < 0.05; ^**^ *p* < 0.01; ^***^ *p* < 0.001

## Discussion

As shown in Fig. 1, the temporal trends for the percentage of patients who underwent LA in both the LIP and GP exhibited a steady growth trend to approximately half of the total appendectomies in 2010–2011 (Fig. 1). The trends indicated that LA has gained wide acceptance in the treatment of appendicitis in Taiwan in recent years. However, the percentage of patients in the LIP who underwent LA was still lower than that in the GP for each year. This phenomenon was consistent with some previous studies indicating that vulnerable patient populations are less likely to receive treatment at institutions with newer technologies [20, 24]. One reason for this finding may be because more LIP patients live in remote areas than GP patients; thus, location renders access to medical centers and regional hospitals more inconvenient for LIP patients than GP patients. We can observe this phenomenon in some of the analytical results. For example, the percentage of LIP patients residing in urban areas was lower than that of GP patients (75.97 % for LIP vs. 85.78 % for GP, *p* < 0.05), and fewer LIP patients were hospitalized in medical centers than GP patients (24.45 % for LIP vs. 34.24 % for GP, *p* < 0.05) [6, 25].

In our analysis, we observed that LIP patients were more susceptible to the NHI payment policy than GP patients. As shown in Fig. 1, the growth rate in 2010 (compared with 2009) was 9.87 % for LIP patients who underwent LA; this value was significantly higher than the growth rates in 2009 (1.64 %) and 2011 (3.41 %), likely because the claims for appendicitis were processed by case payment before December 31, 2009. Thus, some of the material costs of LA were not included in the scope of the NHI payment. This portion of hospital expenses may require payment by the patients themselves, which led to some LIP patients not selecting LA due to economic reasons. Fortunately, the payment claims for appendicitis were changed to Taiwan Diagnosis Related Groups (Tw-DRGs); since January 1, 2010, all LA costs have been included in the NHI payment, indicating that the change in the NHI payment policy had a significant impact on the selection of LA in LIP patients. However, the impact of the change in the NHI payment policy on GP patients was not as obvious as it was in LIP patients; we can observe that the growth rate in 2010 was not higher than the growth rates in 2009 and 2011 (8.30 % for 2009, 5.08 % for 2010, and 6.14 % for 2011) in GP patients (Fig. 1). We also observed obvious growth rates for LIP patients who underwent LA in 2007 and 2008 (9.23 % in 2007, and 10.45 % in 2008). This finding is attributable to the fact that although the NHI program was founded in 1995, reimbursement for LA was not available until 2007 [17]. This policy also led to a dramatic increase in GP patients who underwent LA in 2007 (12.36 %), but the growth rate stabilized in 2008 (6.79 %).

Although LA is a standardized operation, the influence of the revision of NHI payment policy was still significant for LIP patients, and we can predict that the effect of the policy will be greater for other more expensive and complex operations. This phenomenon indicated that there are still disparities in the selection of treatment type between the LIP and GP, and equal access to health care does not eliminate disparities. Thus, the formulation and revision of NHI policies must consider not only the fairness of the system itself for all patients but also the equality of opportunity for choice for vulnerable populations. We must avoid a situation in which the same disease has different medical decisions regarding treatment type between the LIP and GP due to the special nature of the LIP as economically disadvantaged. We suggest that in the process of formulating and revising NHI policies, governmental and related agencies should pay more attention to the supporting standards and procedures for vulnerable populations to achieve a balance between government capacity and the equality of opportunity for choice in medical decisions for all patients. Moreover, we hope that there will be more research focusing on the equality of opportunity in medical decision choices for vulnerable populations in the future.

Figure 2 presents an interesting phenomenon: younger patients (0- to 29-year-old age group) were more likely to choose LA over OA compared with relatively older patients (aged 30 years and older). This finding may be because young people are more willing to try new approaches; although LA is not new, OA is more conventional than LA. In contrast, older patients may be more conservative and more inclined to adopt traditional operation methods. In addition, the risk of complications may increase with age; thus, some physicians and patients may be more inclined to choose the conservative operation type. Compared with OA, LA was associated with a slightly higher rate of routine discharge of patients (99.67 % after LA, 98.07 % after OA) and a lower rate of in-hospital complications (1.48 % after LA, 3.76 % after OA). The rate of readmission for complications was lower in patients who underwent LA than in patients who underwent OA (1.64 % vs. 3.89 %); this finding concurs with the results of prior studies [26, 27]. In addition, we observed that the overall case-fatality rate of patients who underwent LA was 0 %, but the case-fatality rate of patients who underwent OA was 0.53 % (Table 3).

Regarding hospital costs and LOS, LIP patients benefit more from the LA approach than they do from the OA approach in the subpopulations of male patients, patients aged 45 years and older, patients with a CCI score of two or more, and patients with complicated appendicitis (Table 5). For instance, the mean LOS for patients who underwent LA was 3.68 and 3.91 days shorter than OA patients in the 45- to 59-year-old and 60 years and older age groups, respectively; however, the corresponding values were only 0.97 and 0.69 days shorter in the 0- to 14-year-old and 15- to 29-year-old age groups, respectively (Fig. 3). Furthermore, the hospital costs for LA were higher than those for OA in the 0- to 44-year-old age group but lower for those aged 45 years or older. In addition, more cost savings were found as age increased (Fig. 4), indicating that older LIP patients benefit more from the LA approach than younger LIP patients in the treatment of appendicitis when costs and LOS are considered. This finding is consistent with the conclusion of one paper [10], which concluded that patients aged 65 years or older, patients with comorbidities, and patients with complicated appendicitis benefit more from the laparoscopic approach for the treatment of appendicitis when considering costs and LOS.

The NHIB has established a uniform system to control the quality of medical services and coding. When the medical services that are provided to beneficiaries by contracted medical care institutions are deemed to be incompatible with the provisions of the NHI Act by the Professional Peer Review Committee, the expenses thereof are borne by the contracted medical care institutions themselves. Otherwise, the Disputes Settlement Board, which was established under the NHI scheme, settles disputes that arise in cases that were approved by the insurer and in cases that were claimed by the insured, group insurance applicants, or contracted medical care institutions [6, 28]. Based on the above parameters, the data acquisition quality of the present study can be considered to be reliable. However, the presented data are still subject to limitations. First, this study is similar to other administrative and claimed database-based studies in that we could not review individual patient medical records that contained clinical data, and all information was in the form of numbers or codes. Without reviewing the individual medical records of each patient to ensure that the records were coded precisely, deviations between the codes and the actual severity of the disease could exist. Second, the dataset used does not contain data on postoperative courses, such as the time to first flatus passage, time to oral intake, or intensity of pain [17]. Finally, some cases of complicated appendicitis should be treated by OA, which may lead to a worse outcome for OA compared to LA and increase the risk of residual confounding factors in our analysis. Nonetheless, because the same database has been applied in many other fields of study with numerous high-impact publications, we believe that this population-based national claims database can be considered reliable [8].

## Conclusions

The present study revealed that LA has gained wide acceptance for the treatment of appendicitis in Taiwan in recent years. However, the percentage of patients who underwent LA in the LIP was still lower than that in the GP in each year. The LIP patients were more affected by the NHI payment policy than GP patients; thus, more attention should be paid to the effects of the formulation and revision of NHI payment policies on vulnerable populations. We also observed that older patients were less likely to undergo LA rather than OA compared with younger patients. LA has significant advantages over OA regarding LOS, in-hospital complications, in-hospital mortality, and rate of routine discharge. Furthermore, regarding hospital costs and LOS, LIP patients benefitted more from the LA approach than from the OA approach in the subpopulations of male patients, patients aged 45 years and older, patients with a CCI score of two or more, and patients with complicated appendicitis. In summary, LIP patients, particularly the elderly, benefit more from the LA approach than the OA approach in the treatment of appendicitis.

## Abbreviations

LA: Laparoscopic appendectomies
OA: Open appendectomies
LIP: Low-income population
GP: General population
NHI: National Health Insurance
NHIB: National Health Insurance Bureau
NHIRD: National Health Insurance Research Database
NHRI: National Health Research Institutes
LOS: Length of hospital stay
CCI: Charlson Comorbidity Index
DD: Inpatient expenditures by admissions
DO: Details of inpatient orders
CD: Ambulatory care expenditures by visits
OO: Details of ambulatory care orders
IRB: Institutional Review Board
CI: Confidence intervals
ANOVA: Analysis of variance
Tw-DRGs: Taiwan Diagnosis Related Groups
